# IL-4/10 prevents stress vulnerability following imipramine discontinuation

**DOI:** 10.1186/s12974-015-0416-3

**Published:** 2015-10-31

**Authors:** Arum Han, Hyelim Yeo, Min-Jung Park, Seung Hyun Kim, Hyun Jin Choi, Chang-Won Hong, Min-Soo Kwon

**Affiliations:** Department of Pharmacology, School of Medicine, CHA University, CHA BIO COMPLEX, 335 Pangyo, Bundang-gu, Seongnam-si, Gyeonggi-do 463-400 South Korea; Cell Therapy Center and Department of Neurology, College of Medicine, Hanyang University, Haengdang-dong, Seoul South Korea; College of Pharmacy, CHA University, CHA BIO COMPLEX, 335 Pangyo, Bundang-gu, Seongnam-si, Gyeonggi-do 463-400 South Korea; Department of Pharmacology, Infectious Disease Medical Research Center, College of Medicine, Hallym University, Chuncheon, 200-702 South Korea

**Keywords:** Stress vulnerability, Depression, IL-4, IL-10, Microglia

## Abstract

**Background:**

Identifying stress vulnerability after antidepressant discontinuation may be useful in treating relapses in depression. Previous studies have suggested significant effects of the immune system as well as the central nervous system (CNS) on progression and induction of major depression. In the present study, we hypothesized that the factors that are not rescued by a tricyclic antidepressant imipramine may be associated with stress vulnerability and relapses in depression.

**Methods:**

To address this issue, mice were exposed to 2 h of restraint stress for 21 consecutive days (chronic restraint stress (CRS)) with or without co-treatment of imipramine. These groups were exposed to an electronic foot shock (FS) as additional stress after imipramine washout. Main targets of stress and antidepressants were analyzed in the hippocampus, lymph node, and serum after a series of depression-like behavior analysis.

**Results:**

In this study, we found for the first time that mice exposed to CRS with a tricyclic antidepressant imipramine co-treatment, which did not show depressive-like behaviors, were vulnerable to subsequent stressful stimuli compared to the non-stressed mice after imipramine washout. CRS mice with imipramine co-treatment did not show any difference in BDNF, serotonin receptors, pro-inflammatory cytokines, or kynurenine pathway in the hippocampus compared to the controls. However, peripheral IL-4, IL-10, and alternatively activated microglial phenotypes in the hippocampus were not restored with sustained reduction in CRS mice despite chronic imipramine administration. Supplementing recombinant IL-4 and IL-10 in co-Imi+CRS mice prevented the stress vulnerability on additional stress and restored factors related to alternatively activated microglia (M2) in the hippocampus.

**Conclusion:**

Thus, our results suggest that the reduced IL-4 and IL-10 levels in serum with hippocampal M2 markers may be involved in the stress vulnerability after imipramine discontinuation, and the restoration and modulation of these factors may be useful to some forms of depression-associated conditions.

**Electronic supplementary material:**

The online version of this article (doi:10.1186/s12974-015-0416-3) contains supplementary material, which is available to authorized users.

## Background

Major depression has various types of symptoms and disease courses with inconsistent response to treatments [1]. More than 50 % of patients, who recover from a first episode of depression, are likely to suffer additional episode of major depression, resulting in a chronic lifelong illness [2, 3]. Stress vulnerability constitutes intrinsic brain abnormality and psychosocial stressful stimuli. Thus, identifying and characterization of factors related to stress vulnerability are crucial in management of major depressive disorders [3].

Involvement of immunology in depression pathophysiology has been a subject of research interest. “Cytokine theory of depression” proposed that cytokines are associated with the pathogenesis of depression by communicating with the central nervous system (CNS) [4, 5]. Numerous studies have reported that circulating pro-inflammatory cytokines, such as interleukin-1 beta (IL-1β), IL-6, interferon gamma (IFN-γ), and tumor necrosis factor alpha (TNF-α), may be associated with some forms of major depression [6]. Indoleamine 2,3-dioxygenase (IDO) has been suggested as a factor linking “cytokine theory” and “monoamine theory” in depression, since it is activated by pro-inflammatory cytokines and metabolizes tryptophan with downstream enzymes, leading to serotonin reduction and neurotoxicity [7, 8]. A tricyclic antidepressant imipramine functions by inhibiting serotonin and norepinephrine reuptake, and it was found to have anti-inflammatory effects [9]. However, inconsistent findings regarding effects of stress on inflammatory cytokines are reported, and inflammation-focused involvement of immunology in depression remains controversial [10].

Anti-inflammatory cytokines also have been known to be involved in the pathophysiology of depression and stress response [4, 11]. Previous studies have suggested that anti-inflammatory cytokines affect depressive behavior as a failure to counterbalance the increased expression of pro-inflammatory cytokines [12, 13]. The ratio of IFN-γ to IL-10 was elevated in depressed patients, and it was normalized after prolonged antidepressant treatment [14, 15]. T cells, such as regulatory T cells (T_reg_), may communicate with the CNS via anti-inflammatory functions during stressful episodes, and impaired T cell function may directly contribute to the depression pathogenesis [16]. These findings support and suggest that anti-inflammatory cytokines with T cells may participate in the common pathway in depression and other psychophysiological activities [13].

As part of the innate immune system within the CNS, microglia was found to be involved in the pathophysiology of depression [17, 18]. Although classification is oversimplified, microglia can exhibit classically activated M1 phenotype or M2 phenotype with alternate activation [19]. In vitro, M1 microglia are induced by lipopolysaccharide (LPS) and express pro-inflammatory cytokines, such as IL-1β, TNF-α, and IL-6 [19, 20]. M2 microglia express CX3CR1, CD200R, and CD206 to show neuroprotective effect and homeostatic maintenance [19, 20]. Previous studies found changes in microglial proliferation and activation in mice that were exposed to chronic restraint stress or social defeat stress [21–23]. However, the relationship between peripheral cytokines and microglial phenotypes has not been elucidated in depression until now.

We found for the first time that stress vulnerability could be induced in the mice that were exposed to chronic restraint stress (CRS) with imipramine co-treatment by acute stressor after imipramine discontinuation. Chronic imipramine co-treatment inhibited stress-related behaviors but failed to normalize the decreased peripheral IL-4 and IL-10 and hippocampal M2 microglia factors. In addition, supplement of IL-4 and IL-10 prevented the stress vulnerability with normalization of M2 microglia factors in the hippocampus. Thus, our results propose potential of IL-4 and IL-10 to dampen elevated vulnerability to stress-related events to achieve more comprehensive treatment of depression.

## Methods

### Experimental animals

Male C57BL/6 mice at age of 7 weeks (Orient Bio Inc. Seoul, Korea) weighing 20–25 g were used for all the experiments. Animals were housed five per cage in a room maintained at 22 ± 0.5 °C with an alternating 12-h light–dark cycle. Food and water were available ad libitum. Animals were allowed to acclimate to the laboratory 1 week before the beginning of the experiments. To reduce variation, all experiments were performed during the light phase of the cycle. All experimental procedures were approved by the Animal Care and Use Committee of the CHA University (IACUC130018).

### Drug treatment and stressful stimuli exposure timeline

#### Experiment 1: restraint stress and imipramine treatment

Animals were randomly assigned to a non-stressed control group or a CRS group, and CRS mice were randomly divided and received either normal saline or imipramine (20 mg/kg) co-treatment (co-Imi+CRS). Imipramine (Sigma-Aldrich, St. Louis, Missouri) was dissolved in physiologic normal saline and was administered intraperitoneally 30 min prior to restraint stress. For restraint stress, the mice were forced into 50 mL Corning tubes with a nose-hole for ventilation, 2 h per day (11:00 AM–1:00 PM). The mice were exposed to restraint stress (2 h/day) for 21 consecutive days (CRS). A series of behavior assays were performed within a week after the discontinuation of CRS and imipramine co-treatment. The hippocampus and the mesenteric lymph nodes were dissected 1 day after behavior assessments for Western blot and qPCR analysis. The whole blood was collected by cardiac puncture.

#### Experiment 2: post-short-term restraint stress and electrical foot shock

After 21 days of restraint stress and imipramine administration, mice had resting period of 5 days to allow the drug to be completely washed out [24]. During the washout period, depressive-like behaviors of mice were assessed using sucrose preference (SP) test, elevated plus maze (EPM), and tail suspension test (TST). After 5 days of resting period, foot shock (FS) was delivered in an electrical FS chamber with a grid floor that is connected to an electric shock generator. Mice received a 0.25-mA electrical current of 1-s duration delivered randomly for 10 times over 10 min [25]. Depressive-like behaviors were assessed again 3 days after electrical FS using SP test, light–dark (LD) box, and forced swim test (FST).

#### Experiment 3: IL-4 and IL-10 combination treatment

Experiment 2 described above is repeated with cytokine treatment during the 5-day washout period. Co-Imi+CRS group was randomly assigned to saline or a combination of recombinant mouse IL-4 and IL-10 (R&D Systems, Minneapolis, USA). For 5 days, mice were treated by ip injection of saline or combination of cytokines (100 ng of IL-10 and 100 ng of IL-4). Mice were then exposed to electrical FS stress (identical procedure as described above), and their depression-like behavior was assessed 3 days after the additional stress exposure. The hippocampus and the mesenteric lymph nodes were dissected 1 day after behavior assessments for further molecular analysis.

### Behavioral testing

Mice were allowed to acclimate to a testing room for at least 30 min before performing the assessments. All assessments were conducted during the light cycle between 9:00 AM and 4:00 PM in a series, one assessment per day. SP test was performed independently. LD test and EPM were done using EthoVision XT9 video tracking system (EthoVision^®^, Version 9 Noldus, Netherlands). TST and FST were conducted by two observers to minimize error.

#### Sucrose preference test

Preference for sucrose solution over drinking water was measured in order to assess anhedonia, which is usually altered in depressed mice [26]. To assess SP, mice were provided with two bottles filled with 1 % sucrose diluted in drinking water or drinking water alone. Animals were acclimatized to two bottle conditions for two consecutive days and were tested for their choice for two additional days. The position of the bottles was interchanged during 4 days of testing. On each test day, the fluid levels were noted. SP was calculated as percentage of sucrose/total fluid consumed.

#### Light–dark exploration

Anxiety-like behavior was measured using EPM and LD by assessing their tendency to avoid bright light and open spaces [27, 28]. The apparatus used in this assessment was a box (30 × 30 × 30 cm) consisting of one brightly lit open chamber connected to a darkened enclosed chamber. The chambers were connected by a small square hole (7 × 7 cm). Mice were placed in the corner of the lit chamber, facing away from the dark chamber, and the number of transitions between the chambers and time spent in the dark chamber were manually measured for 10 min. The times spent in each room were recorded using EthoVision XT9 video tracking system (EthoVision^®^, Version 9 Noldus, Netherlands).

#### Elevated plus maze

EPM apparatus and procedure are modified from the original protocol [28]. The apparatus consisted of four open roof arms (30 × 5 cm) made of white matte Plexiglass. The two opposite arms were enclosed with 20-cm-high walls, and the remaining two opposite arms had no walls. The four arms were placed at 90° to each other around a 5 × 5-cm square in the center. The apparatus was elevated 30 cm above the floor. The mouse was initially placed in the center of the apparatus, facing one of the open arms away from an experimenter, and was allowed to explore the apparatus freely for 5 min. The number of entries to open arms and closed arms was recorded, and the times spent in each arms were recorded using EthoVision XT9 video tracking system (EthoVision^®^, Version 9 Noldus, Netherlands).

#### Tail suspension test

The immobility induced by tail suspension was modified from a method described by Steru et al. [29]. The apparatus consisted of a cupboard with a hook attached to the top. The mice were suspended by securing the tail to the hook by wrapping adhesive tape around the tail. The tail was suspended carefully not to fold the tail, and the tip of the tail was wrapped 2 cm away from the top of the hook. The data of the mice that climbed their tails were removed from the test. The time spent immobile during a 7-min testing period was measured. The observers were blinded to the groups. The time spent immobile was measured and compared by two observers to minimize the bias.

#### Forced swimming test

In FST, we assessed the ability of mice to cope with an inescapable stressful situation, which reflects depressive-like behavior [30]. Mice were individually placed in a 2-L Pyrex beaker (13 cm diameter, 24 cm height), filled with 23 °C water with a depth of 17 cm. All mice were forced to swim for 6 min, and the duration of immobility was measured during the final 5 min of the test. The immobility was defined as the time that the mouse spent floating without struggling and making only the movements necessary to keep its head above the water level. The observers were blinded to the groups. The time spent immobile was measured and compared by two observers to minimize the bias.

### Determination of glucocorticoid level

CRS mice were immediately sacrificed after the last restraint stress, and whole blood was collected by cardiac puncture, and serum was isolated and stored at −80 °C until assayed. Serum glucocorticoid level was determined by corticosterone EIA kit according to manufacturer’s instructions (Cayman Chemical, Ann Arbor, MI).

### Total protein extraction and Western blot analysis

Hippocampal protein was extracted and expression levels were assessed using Western blotting. After dissecting the hippocampus, the tissue was washed two times with cold Tris-buffered saline (TBS; 20-mM Trizma base and 137 mM NaCl, pH 7.5). Immediately after washing, cells were lysed with SDS lysis buffer (62.5-mM Trizma base, 2 % *w*/*v* SDS, 10 % glycerol) containing 0.1 mM Na3VO4, 3 mg/ml aprotinin, and 20 mM NaF. After brief sonication to shear DNA and reduce viscosity, the concentration of protein was determined with a detergent-compatible protein assay reagent (Bio-Rad Laboratories) using bovine serum albumin as the standard. After adding dithiothreitol (5 mM) and bromophenol blue (0.1 % *w*/*v*), the proteins were boiled, separated by electrophoresis in 10–16 % polyacrylamide gels (Invitrogen), and transferred onto a polyvinylidene difluoride (PVDF) membrane (Bio-Rad Laboratories). Membranes were blocked on a shaker for 1 h at room temperature. Blocking buffer consisted of TBST (Tris-buffered saline/0.1 % Tween-20) and 5 % skim milk. Primary antibodies were dissolved in the blocking buffer and the membranes were immunoblotted with antibodies against brain-derived neurotrophic factor (BDNF; 1:1000, Abcam), serotonin receptor 1A (5-HT_1A_; 1:1000, Santa Cruz) and serotonin receptor 2A (5-HT_2A_; 1:1000, Santa Cruz), IDO (1:500, Abcam), CX3CR1 (1:400, Abcam), and beta-actin (1:1000, Cell Signaling). The membranes were incubated in the anti-rabbit (1:2000, Cell Signaling) or anti-goat IgG secondary antibodies (1:2000, Santa Cruz) dissolved in the blocking buffer at a room temperature for an hour. The membranes were visualized with ECL-plus solution (Amersham Pharmacia Biotech). Then, the membranes were then exposed to chemiluminescence (LAS-4000, Fujifilm) for detection of light emission. Western blot results were scanned on a densitometer and quantified using MultiGauge (Fujifilm).

### Quantitative reverse transcriptase polymerase chain reaction (qRT-PCR)

The whole hippocampus and the mesenteric lymph nodes were dissected a day after behavior assessments for experiments 1 and 3. For RNA extraction, the frozen hippocampus or mesenteric lymph node was homogenized in 1 mL of QIAzol reagent per 100 mg of tissue (Qiagen, Valencia, CA). Chloroform was added to separate the phase that contains RNA, and isopropyl alcohol was added to precipitate RNA. The precipitated RNA pellet was re-dissolved in DEPC-treated water (Bioneer, Seongnam, Korea) after air-drying the pellet. Quantification of RNA concentration was determined by the absorption at 260 nm. One microgram of messenger RNA (mRNA) was reverse-transcribed into cDNA in 20 μL of reaction mix using Maxime RT PreMix Kit (Intron, Seongnam, Korea). Quantitative PCR was performed using AccuPower GreenStar qPCR PreMix (Bioneer, Seongnam, Korea). Primer sequences are listed in Table 1. The cyclic conditions consisted of an initial enzyme activation at 95 °C for 5 min followed by 40 cycles of denaturation at 95 °C for 20 s, annealing, and extension including detection of SYBR Green bound to PCR product at 56 °C for 40 s. Glyceraldehyde 3-phosphate dehydrogenase (GAPDH) was used as an internal control for normalization. The relative quantities of PCR fragments were calculated using the comparative CT method.

**Table 1:** List of primers that were used in qRT-PCR

	Forward (5′–3′)	Reverse (5′–3′)
GAPDH	CATGGCCTTCCGTGTTCCTA	GCGGCACGTCAGATCCA
BDNF	TGCAGGGGCATAGACAAAAGG	CTTATGAATCGCCAGCCAATTCTC
IDO	GGCTTCTTCCTCGTCTCTCTATTG	TGACGCTCTACTGCACTGGATAC
KAT2	GTTCTCCACACACAAGTCTC	GGATCCATCCTGTCAGTCA
KMO	CCTGTAGAGGACAATATAGGATCAACAA	GCAAGCCCCATCTACTGCAT
5-HT_1A_	CTGTTTATCGCCCTGGATG	ATGAGCCAAGTGAGCGAGAT
5-HT_2A_	CCGCTTCAACTCCAGAACCAAAGC	CTTCGAATCATCCTGTACCCGAA
GATA3	AGAACCGGCCCCTTATCAA	AGTTCGCGCAGGATGTCC
TBX21	AGGGAACCGCTTATATGTCC	TCTCCATCATTCACCTCCAC
FOXP3	GAACCCAATGCCCAACCCTAG	TTCTTGGT TTTGAGGTCAAGGG
RORγ	CCTGGGCTCCTCGCCTGACC	TCTCTCTGCCCTCAGCCTTGCC
IL-1β	GGCTGGACTGTTTCTAATGC	ATGGTTTCTTGTGACCCTGA
IL-4	ACAGGAGAAGGGACGCCATG	GCAGCTTATCGATGAATCCA
IL-6	CCACTTCACAAGTCGGAGGCTTA	GCAAGTGCATCATCGTTGTTCATAC
IL-10	CCAGTTTTACCTGGTAGAAGTGATG	TGTCTAGGTCCTGGAGTCCAGCAGACTCAA
IL-13	TGGGTCCTGTAGATGGCATTG	AGACCAGACTCCCCTGTGCA
IL-17	CTCCAGAAGGCCCTCAGACTAC	GGGTCTTCATTGCGGTGG
IFN-γ	TGA ACG CTA CAC ACT GCA TCT TGG	CGA CTC CTT TTC CGC TTC CTG AG
TNF-α	GAGTCCGGGCAGGTCTACTTT	CAGGTCACTGTCCCAGCATCT
CD200R	AAATGCAAATTGCCAAAATTAGA	GTATAGCTAGCATAAGGCTGCATTT
CD206	TCTTTGCCTTTCCCAGTCTCC	TGACACCCAGCGGAATTTC
CX3CR1	CAGCATCGACCGGTACCTT	GCTGCACTGTCCGGTTGTT
NOX2	GACCCAGATGCAGGAAAGGAA	TCATGGTGCACAGCAAAGTGAT

### Serum cytokine assay

Serum cytokine levels were measured using Bio-Rad Bio-Plex^®^ assay (Bio-Rad, Hercules, CA). The lower detection limits of IL-1β, IL-4, IL-6, IL-10, IL-17, TNF-α, and IFN-γ were 9.4, 2.1, 0.2, 1.0, 0.8, 1.2, and 1.4 pg/mL, respectively. The cytokine levels were measured and analyzed according to the manufacturer’s instruction.

### Statistical analysis

Data were presented as the mean ± standard mean error (SEM). The statistical significance of differences between groups was assessed with Student’s *t* test and one-way or two-way analysis of variance (ANOVA) using GraphPad Prism version 5.0 for Mac (GraphPad, La Jolla, CA). Bonferroni’s post hoc analysis was performed when *p* values were <0.05.

*p* < 0.05 was considered as statistically significant.

## Results

### CRS-induced depression-like behaviors and chronic imipramine treatment reversed the depressive-like behaviors

**Fig. 1:**
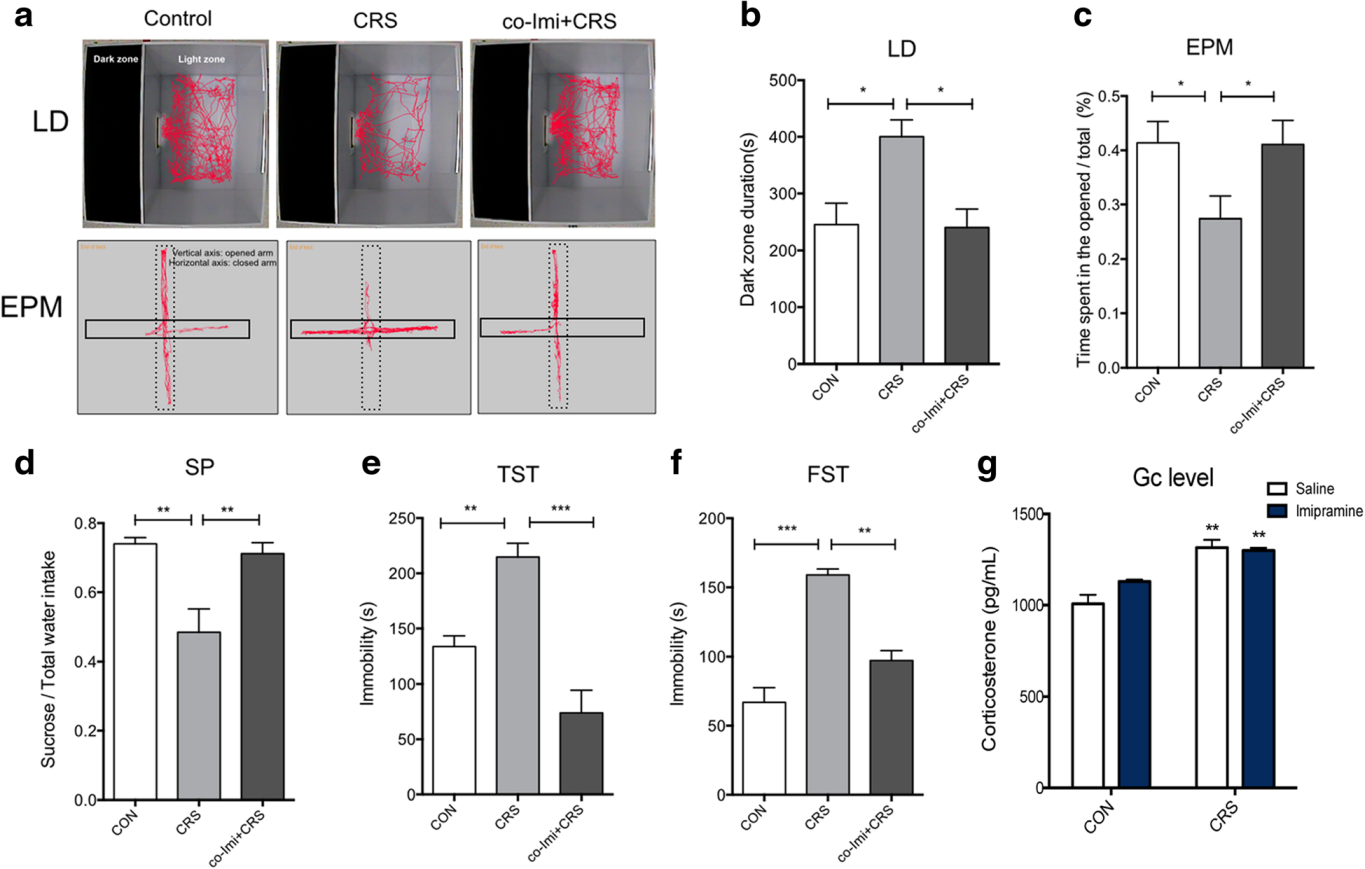
Chronic restraint stress-induced anxiety-like and depressive-like behaviors and imipramine co-treatment prevented the induction of stress-related behaviors. Mice were exposed to restraint stress for 21 days (CRS) with saline or imipramine (20 mg/kg; co-Imi+CRS). A serial of behavioral assessments were finished within a week after the termination of CRS and imipramine co-treatment. Anxiety-like behaviors, such as light–dark (LD) box and elevated plus maze (EPM), were assessed using EthoVision XT9 (**a**). *LD* the time spent in the dark zone during the 10 min was measured (**b**). *EPM* the time spent in the open arms was measured and was expressed as a percentage (**c**). Sucrose preference (*SP*) for 1 % sucrose solution over regular drinking water was examined for 2 days after 2 days of inhabitation to two bottle conditions (**d**). Tail suspension test (*TST*) was done for a 7-min period, and the duration that the subject remained immobile was calculated (**e**). Forced swimming test (*FST*) was done for 5 min following a minute of pre-swimming, and the total immobility time after the pre-swimming was measured (**f**). *p < 0.05, **p < 0.01, ***p < 0.001 compared with the controls unless indicated otherwise with an arrow, *n* = 10–13 in each group and the data shown are mean ± standard mean error (SEM). Serum glucocorticoid level (*Gc level*) was analyzed immediately after CRS by performing enzyme-linked immunosorbent (EIA) assay (**g**). **p < 0.01 compared with the controls, *n* = 5 in each group and the data shown are mean ± standard mean error (SEM)

Animals were exposed to CRS in reference to the previous study that reported changes in behaviors and molecular mechanisms that are relevant to depression [31, 32]. In LD, CRS mice spent more time in the dark compared to the controls, and imipramine treatment reduced the time spent in the dark (Fig. 1a, b). Similarly, CRS mice exhibited lower percentages of the time spent in the open arms than the controls and spent more time in the closed arms in EPM (Fig. 1a, c). Moreover, CRS mice exhibited a significant decrease in SP compared to the controls (Fig. 1d). Imipramine co-treatment to CRS mice (co-Imi+CRS) prevented the decrease in SP and normalized the preference similar to that of the controls. In both TST and FST, exposure to CRS increased immobility compared to the controls, suggesting that CRS mice exhibited depression-like behaviors. Imipramine treatment significantly decreased the immobility time, and this shows that imipramine was successful to prevent behavioral despair induced by CRS in both TST and FST (Fig. 1e, 1f). Imipramine treatment to the controls did not affect stress-related behavior (Additional file 1: Figure S1). The glucocorticoid (Gc) level was elevated immediately after termination of CRS (*F*_1, 20_ = 33.60, *p* = 0.0004), which suggests that habituation did not occur in response to the chronic restraint procedure used in the current study. Chronic imipramine treatment did not alter Gc levels in the controls and in CRS mice, and this indicates that imipramine does not affect HPA axis activation (Fig. 1g). Collectively, 3 weeks of restraint stress induced significant changes in behaviors that are associated with the characteristics of chronic psychological stress, and imipramine co-treatment prevented induction of stress-associated behaviors.

### CRS mice with imipramine co-treatment exhibited depressive-like behaviors when exposed to a short-term acute stress after imipramine washout

**Fig. 2:**
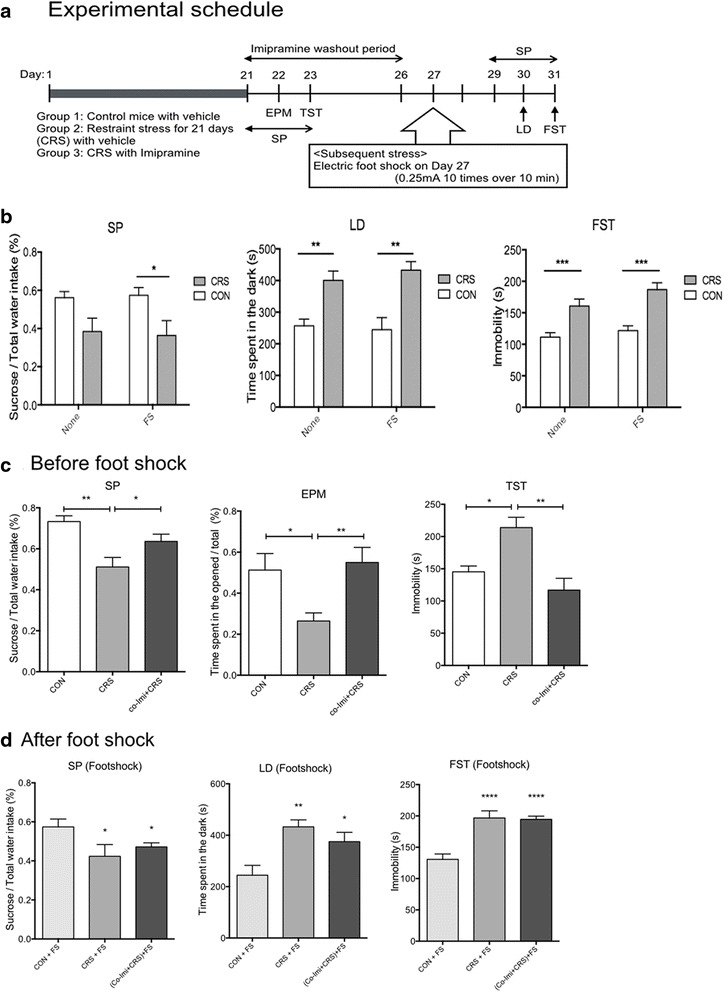
CRS mice with imipramine co-treatment exhibited anxiety-like and depressive-like behaviors upon an exposure to short-term acute stress. Drug treatment and stressful stimuli exposure timeline: *CRS*, chronic restraint stress; *SP*, sucrose preference test; *EPM*, elevated plus maze; *TST*, tail suspension test; *RS*, restraint stress; *LD*, light–dark box; *FST*, forced swimming test (**a**). Depressogenic effect of foot shock stress was assessed, and foot shock stress alone does not affect anxiety-like or depressive-like behaviors in the non-stressed (*CON*) and *CRS* mice (**b**). Prior to the exposure to foot shock, CRS mice with imipramine co-treatment (*co-Imi+CRS*) did not exhibit anhedonic behavior (sucrose preference test, *SP*), anxiety-like behaviors in *EPM*, and depressive-like behaviors in *TST* compared with CON (**c**). However, following the exposure to electric foot shock, they (*co-Imi+CRS*) showed decreased sucrose preference, exhibited anxiety-like behavior in *LD*, and depressive-like behaviors in *FST* compared with CON (**d**). *p < 0.05, **p < 0.01, ***p < 0.001, ****p < 0.0001 compared with the controls unless indicated otherwise with an arrow, *n* = 8–10 in each group and the data shown are mean ± standard mean error (SEM). Three times independent experiments was performed, giving a total of nine per group in the final analysis for Western blot

Co-Imi+CRS was further assessed for its vulnerability for depressive-like symptoms when exposed to an additional stressor after termination of CRS and imipramine. The experimental schedule is described in Fig. 2a. FS stress was assessed as an additional stressor. The controls and the CRS mice were divided into two groups, and one group received FS and the other group was put in the shock chamber for 10 min without electrical stimuli. As shown in Fig. 2b, CRS was found to have a significant effect and increased stress-associated behaviors in all behavior assays (SP, *F*_1, 20_ = 12.21, *p* = 0.0023; LD, *F*_1, 32_ = 24.41, *p* < 0.0001; FST, *F*_1, 41_ = 36.60, *p* < 0.0001). However, FS itself in controls and CRS mice did not have any significant effect on stress-associated behaviors in all of the behavior assays (SP, *F*_1, 20_ = 0.005616, *p* = 0.9410; LD, *F*_1, 32_ = 0.09017, *p* = 0.7659; FST, *F*_1, 41_ = 3.697, *p* < 0.0615).

After confirming that FS did affect behavior assay in controls and CRS mice, preliminary behavioral tests were done during the imipramine washout period and confirmed the effects of CRS and imipramine (Fig. 2c). Imipramine washout itself did not induce depression-like behaviors in co-Imi+CRS (data not shown). The controls, CRS, and co-Imi+CRS mice were exposed to FS (CON+FS, CRS+FS, and (co-Imi+CRS)+FS, respectively), and depression-associated behaviors were assessed. After FS exposure, both CRS+FS and (co-Imi+CRS)+FS showed depression-like behaviors in SP, LD, and FST while CON+FS did not (Fig. 2d). When 3 days of RS was introduced as an additional stressful stimulus, it also induced depression-like behaviors in all groups, except for the controls without previous stress exposure (data not shown). These results indicate that co-Imi+CRS mice exhibit both anxiety-like just like CRS and depression-like symptoms when exposed to FS with imipramine washout, even though they did not exhibit depressive-like behaviors before. In sum, co-Imi+CRS mice seemed to be more vulnerable to additional stressful stimuli compared to the controls after imipramine washout, and this implies that chronic imipramine treatment was not successful to normalize the detrimental effects of CRS comprehensively.

### Imipramine co-treatment could not normalize M2 microglia factors in the hippocampus of the CRS

**Fig. 3:**
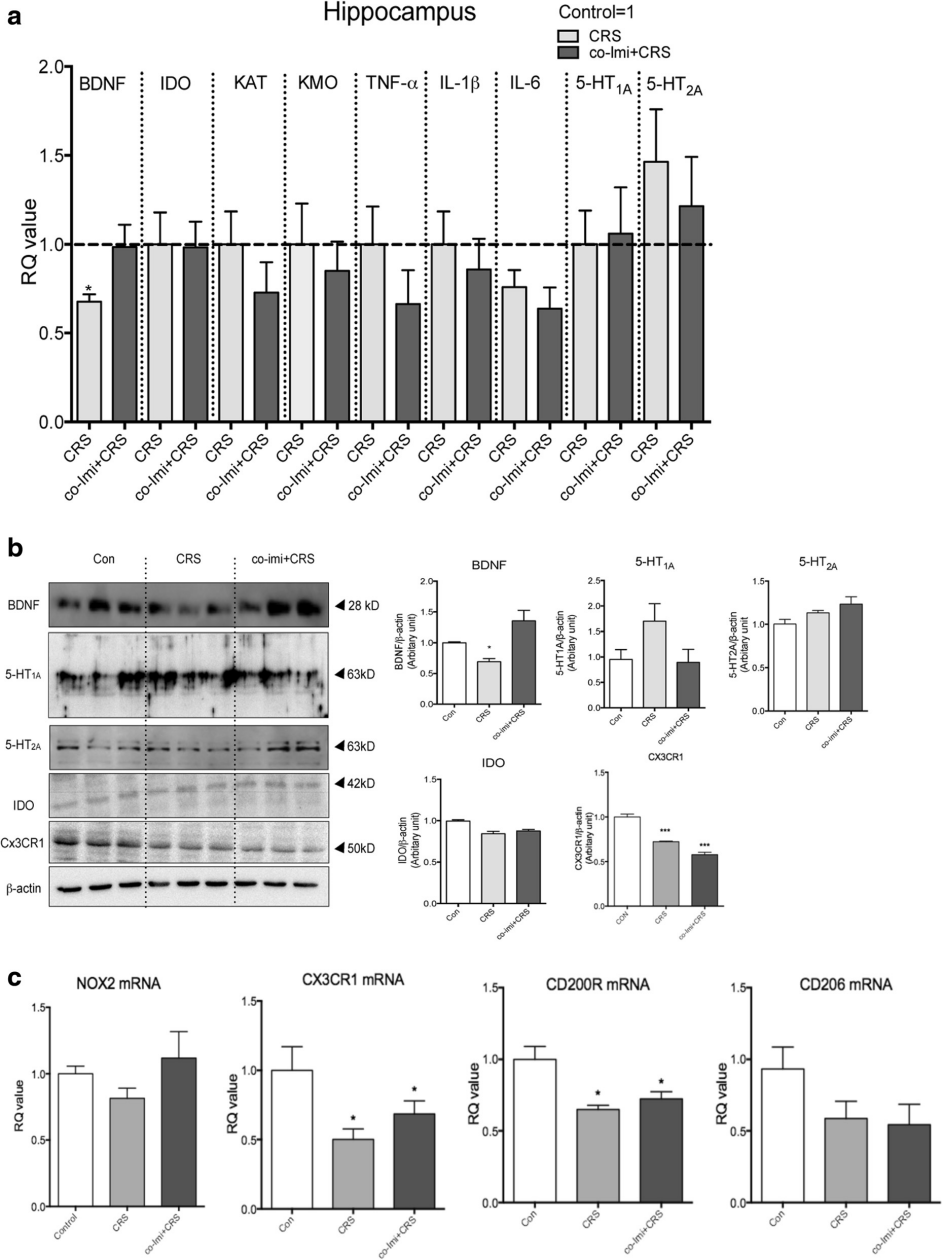
Depression-related factors in the hippocampus were normalized by imipramine co-treatment while M2-related microglia phenotypes were not rescued by imipramine. CRS was conducted for 2 h/day for 21 consecutive days by placing the mouse in a 50-mL Corning tube, and 20 mg/kg of imipramine was administered intraperitoneally 30 min prior stress. The mice were sacrificed 1 day after Fig. 1 of behavior assessment. The hippocampi were dissected for quantitative reverse transcriptase polymerase chain reactions (**a**, **c**) and Western blot (**b**) analysis. Quantitative reverse transcriptase polymerase chain reactions were done using mRNA extracted from the mouse hippocampus (*n* = 10–15). The CT values were normalized as a ratio as controls being 1, which is indicated by *dotted lines* (**a**). Western blot analysis was done using 50 μg of total protein extracted from the mouse hippocampus (*n* = 3). Expressed BDNF, serotonin receptor 1A (*5-HT*_*1A*_), serotonin receptor 2A (*5-HT*_*2A*_), *IDO*, *CX3CR1*, and *β-actin* were quantified using MultiGauge application (Fujifilm) (**b**). Changes in mRNA of NADPH oxidase (*NOX2*) and M2 microglia markers, such as fractalkine receptor (*CX3CR1*), CD200 receptor (*CD200R*), and *CD206*, were measured (*n* = 7–10). *RQ value* refers to the ratio of respective factors as a percentage of the controls (**c**). The data shown are mean ± standard mean error (SEM) *p < 0.05, ***p < 0.001 compared with the controls unless indicated otherwise with an arrow

Based on the hypothesis that the factors that are not rescued by antidepressant may be associated with stress vulnerability, we examined the main targets of stress and antidepressant in the hippocampus using qPCR and Western blot. As shown in Fig. 3a, b, BDNF was reduced significantly in CRS mice and was reversed by imipramine co-treatment, and this change was confirmed by Western blotting. Although we examined that mRNA levels of TNF-α, IL-6, KMO, and KAT were increased within a short time (6 h) after the final restraint stress session, the mRNA levels of IDO, kynurenine pathway enzymes (KAT and KMO), TNF-α, IL-1β, and IL-6 were not altered by CRS mice or imipramine co-treatment which was analyzed after behavior assessments (at least 7 days after final restraint stress session) (Fig. 3a). 5-HT_1A_, 5-HT_2A_ and IDO expression did not show a difference across the groups (Fig. 3a, b).

Since it has been reported that chronic stressful stimuli may contribute to changes in hippocampal microglial effector phenotypes [10, 33], we examined the changes in hippocampal microglial phenotype markers. Immunoreactivity of ionized calcium-binding adaptor molecule-1 (Iba-1), a microglial marker, did not show a significant difference in co-Imi+CRS mice compared to the controls (data not shown). There was no difference in NADPH oxidase (NOX2), an M1 microglia marker, across the groups (Fig. 3c). However, M2-related markers, CX3CR1 and CD200R, were decreased in the hippocampus of the CRS mice, and chronic imipramine treatment was not successful to reverse this decrease (Fig. 3c). CD206, another M2 microglia marker, also showed the same trend as CX3CR1 and CD200R but lacked statistical significance (Fig. 3c). Our results suggest that imipramine can normalize the hippocampal factors that were traditionally considered to contribute to depression in all groups. However, the reduced M2-related markers in CRS mice were not normalized by the imipramine treatment. These results suggest that vulnerability to additional stressful stimuli may be due to M2-related factors, not the usual depression-related factors.

### Imipramine co-treatment did not prevent sustained reduction of IL-4 and IL-10 levels in the lymph nodes and the serum of CRS

**Fig. 4:**
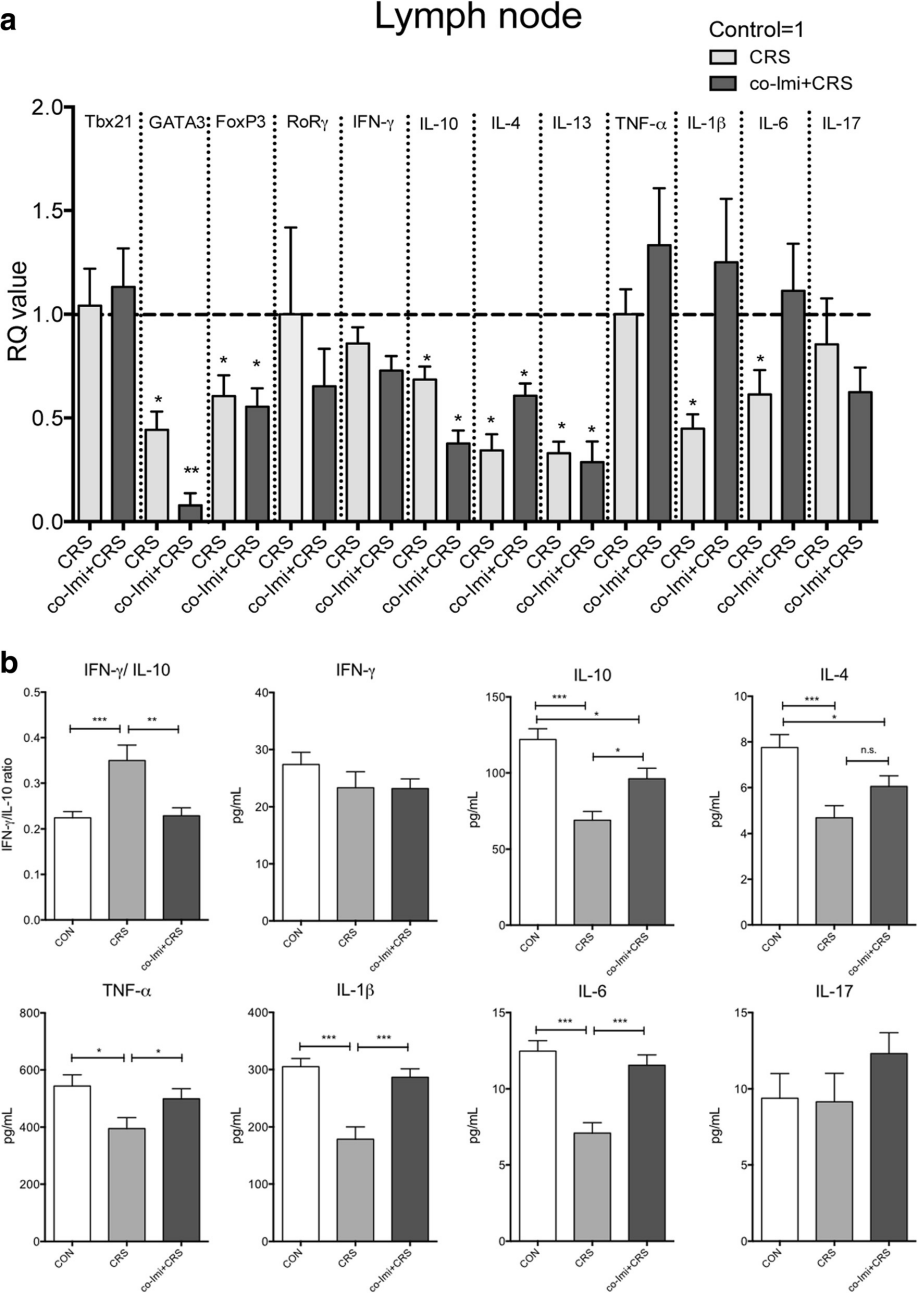
The reduced IL-4 and IL-10 with Th2- and T_reg_-related transcription factors were not rescued by imipramine treatment. CRS was conducted for 2 h/day for 21 consecutive days by placing the mouse in a 50-mL Corning tube, and 20 mg/kg of imipramine was administered intraperitoneally 30 min prior stress. The mice were sacrificed 1 day after Fig. 1 of behavior assessment. The mesenteric *lymph nodes* (**a**) were dissected for quantitative reverse transcriptase polymerase chain reactions and serum (**b**) was obtained to measure circulating levels of pro-and anti-inflammatory cytokines using Bio-Rad Bio-Plex^®^ assay. The CT values were normalized as a ratio as controls being 1, which is indicated by *dotted lines. RQ value* refers to the ratio of respective transcription factors as a percentage of the controls. *p < 0.05, **p < 0.01, ***p < 0.001 compared with the controls, *n* = 10–15 per group and the data shown are mean ± standard mean error (SEM)

The immune system is reported to be involved in the depression pathophysiology and stress responses [4, 34]. As shown in Fig. 4a, mRNA levels of forkhead box P3 (FOXP3; a T_reg_-specific transcription factor) and GATA3 (a Th2-specific transcription factor) were decreased in CRS mice, and imipramine co-treatment did not reverse the reduced FOXP3 and GATA3 mRNA. GATA3 appears to be decreased in Co-imi+CRS compared to the CRS mice but lacked statistical significance. T box 21 (TBX21; a Th1-specific transcription factor) and RAR-related orphan receptor gamma (RORγ) expression, a transcription factor of Th17, remained constant in both the controls and CRS mice. The mRNA levels of IL-4, IL-10, and IL-13 were significantly decreased in CRS and not rescued by imipramine co-treatment. IL-1β and IL-6 levels also were decreased but rescued by imipramine co-treatment. IFN-γ, TNF-α, and IL-17 did not exhibit any difference across all groups (Fig. 4a).

To confirm whether the alteration of cytokine mRNA in the lymph nodes is related with the serum level, the circulating cytokines IL-4, IL-10, IFN-γ, TNF-α, IL-1β, IL-6, and IL-17 were measured by Bio-Plex^®^ cytokine assay, and we confirmed the immunomodulatory effect of imipramine in CRS mice. The ratio of IFN-γ to IL-10 was calculated using the level measured in this assay and showed a significant increase in CRS, and imipramine treatment reversed this increase (Fig. 4b). Serum IL-4 and IL-10 levels were reduced in CRS mice, and IL-4 level was not reversed by imipramine (Fig. 4b). IL-10 level was reversed partially by chronic imipramine co-treatment, and it was still reduced in co-Imi+CRS mice compared to the controls (Fig. 4b). TNF-α, IL-1β, and IL-6 were significantly decreased by CRS but were restored by imipramine co-treatment (Fig. 4b). There was no significant change in IL-17 upon exposure to CRS or co-Imi+CRS. The change in serum cytokines was similar to the change observed in the lymph nodes. These data indicate a possible association of stress vulnerability with sustained reduction in anti-inflammatory cytokines with reduced levels of T_reg_ and Th2 transcription factors despite the prolonged antidepressant treatment.

### IL-4 and IL-10 supplements prevented vulnerability to subsequent stressful stimuli in CRS mice with imipramine co-treatment, preventing the induction of depression-like behaviors

**Fig. 5:**
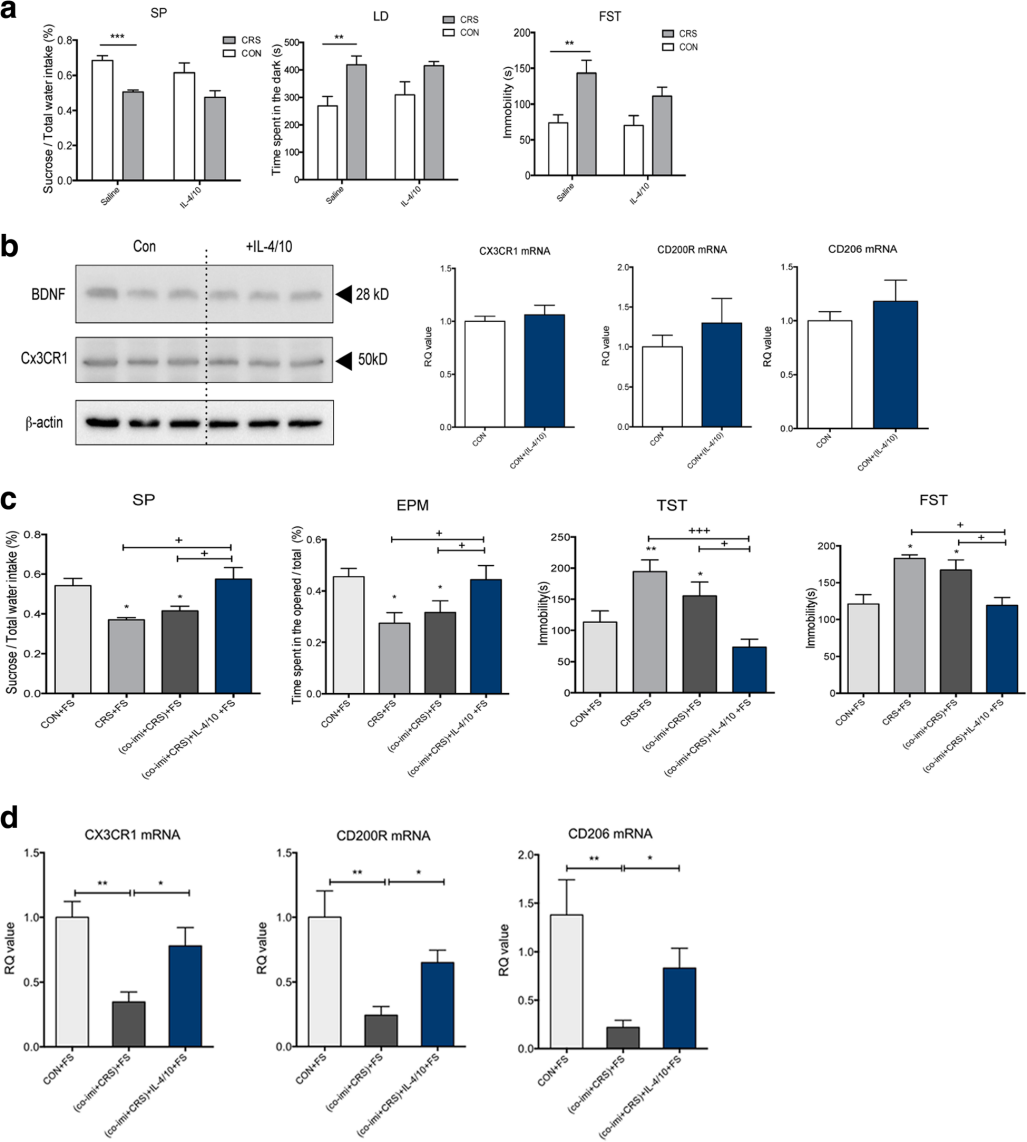
Supplement of IL-4 and IL-10 prevents stress vulnerability following imipramine discontinuation with restoration of type 2 microglia marker messenger RNAs. A combination of IL-4 (100 ng) and IL-10 (100 ng) for 5 days was treated intraperitoneally in non-stressed mice (controls) and CRS mice. A combination of IL-4 (100 ng) and IL-10 (100 ng) for 5 days itself does not have an antidepressant-like effect in controls or CRS mice (**a**). The combination of IL-4 and IL-10 for 5 days does not alter the depression-associated factors, BDNF, and M2-related microglia factor, CX3CR1, in the hippocampus (**b**). Effect of IL-4 and IL-10 in preventing stress vulnerability and depressive-like behaviors of co-Imi+CRS mice was assessed using sucrose preference (*SP*) test, elevated plus maze (*EPM*), tail suspension test (*TST*), and forced swimming test (*FST*) (**c**). Changes in M2 microglia marker expressions, such as fractalkine receptor (*CX3CR1*), CD200 receptor (*CD200R*), and *CD206*, were measured 1 day after (**c**) of behavior assays (**d**). *RQ value* refers to the ratio of respective factors as a percentage of the controls. +*p* < 0.05, +++*p* < 0.001 compared with the groups indicated by an arrow. *p < 0.05, **p < 0.01, ***p < 0.001 compared with the controls, *n* = 12–15 per group and the data shown are mean ± standard mean error (SEM)

Since the levels of IL-4 and IL-10 remained decreased despite the chronic imipramine treatment, we supplemented 100 ng of each mouse recombinant IL-4 and IL-10 to co-Imi+CRS mice to assess whether immune balance by these supplements can prevent the induction of depression-like behaviors upon an exposure to an additional stressful stimulus. First, antidepressant effect of combination of IL-4 and IL-10 (IL-4/10) themselves was assessed at the dose. The controls and the CRS mice were divided into two groups, and one group was injected with saline and the other group was injected with 100 ng of each IL-4 and IL-10 for 5 days. As shown in Fig. 5a, CRS mice showed depressive-like behaviors (SP, *F*_1, 23_ = 16.40, *p* = 0.0005; LD, *F*_1, 32_ = 13.88, *p* = 0.0008; FST, *F*_1, 30_ = 14.49, *p* = 0.0006). However, IL-4/10 treatment itself did not have any significant effect on non-stressed (controls) or CRS mice in the behavior assays (SP, *F*_1, 23_ = 1.636, *p* = 0.2136; LD, *F*_1, 32_ = 0.2902, *p* = 0.5938; FST, *F*_1, 30_ = 1.499, *p* = 0.2304). IL-4/10 treatment did not affect BDNF and CX3CR1 expression in Western blot and has no change in mRNA levels of CX3CR1, CD200R, and CD206 (Fig. 5b, c). These data indicate that the combination of IL-4 and IL-10 itself did not have an antidepressant effect at this dose.

After confirming the absence of antidepressant effect of IL-4/10, we investigated whether IL-4/10 prevents stress vulnerability in co-Imi+CRS mice. During the 5 days of resting period after the termination of CRS, co-Imi+CRS mice were randomly divided into two groups, and each group received saline or IL-4/10. After the cytokine treatment, the mice were exposed to an episode of electric FS, and depressive-like and anxiety-like behaviors were assessed. In all behavioral assays (SP, EPM, TST, and FST), (co-Imi+CRS)+FS mice with saline injection exhibited similar depression-like behaviors to CRS+FS mice (Fig. 5c). However, we found that IL-4/10 alleviated depression-like behaviors that were observed in (co-Imi+CRS)+FS mice (Fig. 5c). Even though the combination of IL-4/10 itself did not have an antidepressant effect, it prevented depression-like behaviors in the co-Imi+CRS mice that were exposed to additional stressor after imipramine washout. These results suggest that promoting peripheral immune balance by supplementing IL-4/10 could be the missing key that imipramine failed to reverse in CRS mice, and they may contribute to lower the vulnerability to additional stressful stimuli.

### M2 microglia phenotype may be associated with IL-4 and IL-10 combination treatment that lowered stress vulnerability

To confirm the correlation between M2 microglia phenotype and IL-4/10 combination treatment, the M2 microglia markers were assessed in the hippocampus of (co-Imi+CRS)+FS mice and their counterparts. As shown in Fig. 5d, the combination of IL-4/10 reversed the reduced mRNA expressions of CX3CR1, CD200R, and CD206 that was not rescued imipramine co-treatment in co-imi+CRS mice with FS exposure. In sum, supplement of IL-4/10 was successful to reverse the change in level of M2 microglia markers, which may be associated with an additional mechanism of IL-4/10 in lowering the vulnerability towards additional stressful stimuli.

## Discussion

Identifying stress vulnerability after antidepressant discontinuation may be useful in treating relapses in depression. In this study, we attempted to find the answer for decreasing vulnerability to additional stressful events in subjects who had previous depressive episode with imipramine discontinuation. We found for the first time that imipramine could not prevent vulnerability to additional stressful stimuli after imipramine discontinuation although imipramine co-treatment to CRS mice inhibited induction of depressive-like behaviors due to its anti-stress effect. The stress vulnerability appears to be related to the decreased serum IL-4 and IL-10 levels despite the imipramine treatment. Thus, we propose that IL-4 and IL-10 may be able to lower the vulnerability to some forms of stress- and depression-related conditions after imipramine discontinuation. In addition, M2 microglia phenotype restoration by IL-4/10 in the hippocampus may be a possible mechanism in lowering the vulnerability towards additional stressful stimuli.

Assuming that the factors that were not recued by imipramine may be involved in the stress vulnerability of co-Imi+CRS mice, we examined the effect of imipramine on alteration of BDNF, serotonin receptors, pro-inflammatory cytokines, components of kynurenine pathway, and microglial phenotype markers in the hippocampus of the mice. BDNF, serotonin receptors, and IDO have been known as main targets of stress and antidepressants in the hippocampus [35, 36]. Microglia are responsible for homeostasis maintenance, neuron support, and neurogenesis, and reports have shown that microglia and neuroinflammation may be involved in depression pathophysiology [10, 19, 37]. In accordance to previous studies [38], BDNF, IDO, and pro-inflammatory cytokine levels did not show significant difference between controls and co-Imi+CRS mice. However, we found that alternative M2 microglia markers, such as CD200R, CX3CR1, and CD206, were reduced in the hippocampus of CRS mice and were not normalized by imipramine treatment. Despite chronic imipramine co-treatment, it could not prevent vulnerability to additional stressful stimuli, and it is speculated to be due to a failure to rescue the M2 microglia phenotype in the hippocampus. This suggests that vulnerability to a subsequent stress may be associated with the changes in M2 phenotypes of microglia, not the main targets of stress and antidepressant in the hippocampus, even though we cannot exclude other brain regions that are also implicated in depression pathophysiology.

Here, imipramine was chosen as an antidepressant, since it has been extensively demonstrated to inhibit the production of pro-inflammatory cytokines and to produce anti-inflammatory cytokines [14, 15, 39]. However, most of these studies were based on ex vivo or in vitro immunological experiments using co-incubation of the whole blood of depressed patients with imipramine so they bear some limitation [9, 14, 15, 40]. In the present study, imipramine co-treatment was successful to normalize the IFN-γ and IL-10 production ratio but could not normalize the absolute levels of IL-4 and IL-10. In addition, transcription factors of Th2 and T_reg_, which are one of the main producers of IL-4 and IL-10 in T cells [41], were not restored by imipramine. Furthermore, stress vulnerability to FS was examined in nude mice, which lacked T cell-mediated immunity, but not in their counterpart mice, Balb/c (Additional file 1: Figure S2). These results suggest that stress vulnerability may be related to impaired T cell function, specifically IL-4 and IL-10 production. Although imipramine co-treatment restored peripheral pro-inflammatory cytokines, such as TNF-α, IL-1β, and IL-6, it was not successful to restore the number of leukocytes reduced by CRS in the blood, spleen, and lymph node (Additional file 1: Figure S3). This result suggests that imipramine may exert immunomodulatory effect by targeting certain subsets of leukocytes. Previous studies have reported that progression and consequence of depression may depend on integrity of the peripheral immune system and chronic stress can impair T cell immunity [42–44]. Additionally, IL-4 and IL-10 are suggested to play a significant role in affecting depression-like behaviors [13, 43, 45, 46]. In the present study, the combination of IL-4 and IL-10 prevented the stress vulnerability in co-Imi+CRS mice that encountered additional stressful stimuli after imipramine discontinuation, even though it did not have an antidepressant effect in the controls or CRS mice. Therefore, we speculated that downregulation of IL-4 and IL-10 may be the impairment in a stress-coping mechanism, which may contribute in developing vulnerability to additional stress-related events.

There are several possible ways that led to changes in microglia by IL-4/10 injection. Chronic physical stress accounts as distress and several reports have reported impaired blood–brain barrier (BBB) permeability in major depressive disorders [47, 48]. Previous study has suggested peripheral immune imbalance is often accompanied by impairment of the BBB, which may allow the entry of IL-4 and IL-10 into the parenchyma [49]. Previous study has found that bone marrow-derived monocytes infiltrated into the paraventricular nucleus upon exposure to chronic psychological stress, which may be associated to regional neuronal and microglial activation [50, 51]. Whether peripheral cytokines reach to parenchyma through the BBB, peripheral cytokine signaling can induce behavioral modification through receptors on the endothelial cells [47, 51, 52]. IL-4 and IL-10 inside the CNS were found to exert its beneficial effect on cognition and memory by alternatively activating microglia (M2) upon interaction with the respective receptors on the microglial cells [19, 53–55]. Previous study has established that CNS microenvironment affects microglial cell function and suggested that microglia’s auto-regulating ability was one of mechanisms for the CNS immunosuppressive state maintenance [53]. The choroid plexus and the cerebrospinal fluid (CSF) environment was found to be an important immunomodulatory gate that can skew immune cells towards alternately activated state due to high levels of the Th2 cells and M2 macrophage skewing cytokines in the CSF [56]. Activated peripheral T cells can shape the CNS by entering the endothelium of the choroid plexus into the cerebrospinal fluid and communicate with the microglia and by modulating and altering the microglial phenotypes [37, 57, 58]. Furthermore, previous studies reported that the peripheral immune system plays a key role in deciding microglial activation and phenotype and can lead to microglial response [54, 59]. Taken together, it is speculated that peripheral administration of IL-4/10 directly or indirectly may affect microglial phenotypes across loosen BBB or through the choroid plexus and blood–CSF barrier (BCSF) or by modulating peripheral immune cells in CRS mice.

Previous research has found a decrease in pro-inflammatory cytokine release and an increase in IL-10 production from LPS stimulated-microglia with imipramine treatment, which partially agrees with what we observed in the hippocampus with CRS [60]. Although several reports examined microglial activation using Iba-1 as target of stress and antidepressants [10, 21–23], normalization of morphological activation marker Iba-1 alone appears to be insufficient to represent and to explain microglial effector function. Recent studies have demonstrated that microglial markers, such as CX3CR1, CD200R, and CD206, have a close relationship in their effector functions [19, 37]. Disturbance of these factors may cause microglia to be non-functional; these factors are suggested to be responsible for pathology of depression [10, 61]. CX3CR1 plays a crucial role in regulating neurotoxicity of microglia, and its stimulation was found to be anti-inflammatory [62, 63]. CX3CR1-deficienct mice showed prolonged depression-like behavior in response to LPS, which suggests that microglial activity may cause a change in stress-related behaviors [62–64]. Especially, the hippocampus was known to have the highest expression of ligand of CX3CR1 and was vulnerable to the loss of CX3CR1 [10, 63]. In our study, CX3CR1 was decreased in CRS, co-Imi+CRS, and (co-Imi+CRS)+FS mice despite chronic imipramine co-treatment in the hippocampus while it was normalized by IL-4/10. Thus, our results suggest that M2 markers may be involved in the mechanism of peripheral immunity affecting CNS. This might be associated with restoring the impairment in stress-coping response and ameliorating the vulnerability to an additional stressor in co-imi+CRS mice after the imipramine discontinuation.

In this study, we examined increased ratio of IFN-γ to IL-10 and decrease in serum TNF-α, IL-1β, and IL-6 levels in CRS mice, and these results appear to be in contrast to most of the previous studies. Many studies have reported increased level of circulating pro-inflammatory cytokines in depression-like conditions [6, 9, 39]. However, there are several studies that reported a decrease or no change in pro-inflammatory cytokines in depressive-like conditions, and they explained this phenomena with hypothalamic–pituitary–adrenal (HPA) axis hyperactivation, which inhibits the secretion of pro-inflammatory cytokines [42, 45, 65–67]. In addition, previous studies suggested that pro-inflammatory cytokines might be a mere characteristic or a nonparticipating factor of depression [46, 65]. In previous studies, dynamic alterations were observed in inflammatory cytokines and microglial function depending on parameters of the stress model [10, 51]. Chronic unrelenting stress (prolonged restraint stress) was found to induce immunosuppression and anti-inflammatory phenotype [68]. Therefore, what we observed in this study may be one of many possible immunological manifestations in major depression. Taken together, it could be postulated that stress vulnerability may be sufficiently induced by reduction in anti-inflammatory cytokines and M2 microglia markers without an increase in pro-inflammatory cytokines. However, the further study will be required to elucidate this hypothesis.

## Conclusion

This study demonstrates that imipramine co-treatment failed to prevent vulnerability to an additional stressor, while it inhibited stress-related behaviors due to its anti-stress effects. Rather than traditionally considered depression-related factors, decrease in the peripheral and hippocampal microglial M2 factors may be involved in the stress-coping system and vulnerability to depressogenic episodes after imipramine discontinuation. The stress vulnerability could be alleviated by supplement of IL-4 and IL-10 in the co-imi+CRS group with reversal in expression of M2 microglia markers. With a further study that attempts to elucidate the underlying mechanism of IL-4 and IL-10 and neuroimmune response by microglia, therapeutic intervention for stress-related disorders might be optimized.

## Additional file

Additional file 1: **Supplementary materials.** S1. Imipramine treatment to naïve mice does not have any effect on depression-like behaviors. S2. BALB/c Nude mice exhibit depressive-like behaviors and anxiety-like behaviors upon brief exposure to acute foot shock stress while balb/c mice with the same genetic background did not. S3. Effect of chronic restraint stress and imipramine treatment on leukocyte counts in the blood, spleen and lymph node. (DOC 255 kb)

## Abbreviations

5-HT_1A_: serotonin receptor 1A
5-HT_2A_: serotonin receptor 2A
BDNF: brain-derived neurotrophic factor
CD200R: CD200 receptor
co-Imi+CRS: CRS mice with imipramine co-treatment
CRS: chronic restraint stress
CX3CR1: fractalkine receptor 1
EPM: elevated plus maze
FOXP3: forkhead box P3
FS: foot shock
FST: forced swim test
HPA: hypothalamic–pituitary–adrenal
IDO: indoleamine 2,3-dioxygenase
IFN-γ: interferon gamma
IL-1β: interleukin-1 beta
IL-4: interleukin-4
KAT: kynurenine aminotransferase II
KMO: kynurenine 3-hydroxylase
LD: light–dark
M1: classically activated type 1 phenotype
M2: alternatively activated type 2 phenotype
NOX2: NADPH oxidase 2
RORγ: RAR-related orphan receptor gamma
SP: sucrose preference
TBX21: T box 21
Th2; T_reg_: regulatory T cells
TNF-α: tumor necrosis factor alpha
TST: tail suspension test
