# Supplementary material for: IL-4/10 prevents stress vulnerability following imipramine discontinuation
Source: J Neuroinflammation. 2015 Oct 31;12:197. doi: 10.1186/s12974-015-0416-3 (PMC4628271; doi:10.1186/s12974-015-0416-3)
Supplement: Supplementary file 1 — Supplementary materials. S1. Imipramine treatment to naïve mice does not have any effect on depression-like behaviors. S2. BALB/c Nude mice exhibit depressive-like behaviors and anxiety-like behaviors upon brief exposure to acute foot shock stress while balb/c mice with the same genetic background did not. S3. Effect of chronic restraint stress and imipramine treatment on leukocyte counts in the blood, spleen and lymph node. (DOC 255 kb) [file 12974_2015_416_MOESM1_ESM.doc]

**Supplementary Materials**


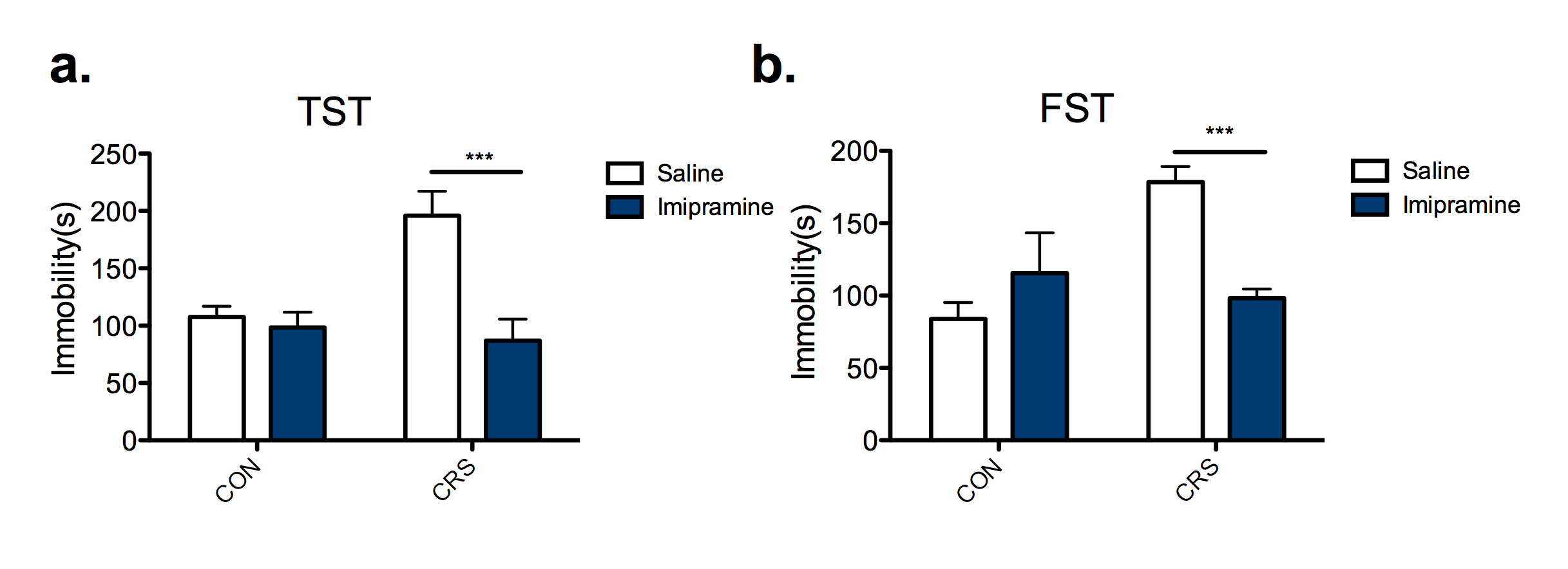


**S1. Imipramine treatment to naïve mice does not have any effect on depression-like behaviors.** The controls and the CRS mice were divided into two groups and one group was injected with saline and the other group was injected with 20mg/kg of imipramine for 21 days. Depressive-like behaviors were measured by tail suspension test (a) and forced swimming test (b). As shown in Figure S1, CRS mice showed depressive like behaviors compared to the controls (TST, F_1, 26_=9.10, p=0.0056; FST, F_1, 31_=16.45, p=0.0003). Imipramine administration to control mice did not affect depression-associated behavior, while it significantly decreased immobility in CRS mice (TST, p=0.001; FST, p=0.001). *** p<0.001, n = 15-20 per group and the data shown are mean ± standard mean error (SEM).

**S2. BALB/c Nude mice exhibit depressive-like behaviors and anxiety-like behaviors upon brief exposure to acute foot shock stress while balb/c mice with the same genetic background did not.** The role of T cells is suggested to be involved in increased vulnerability to stressful stimuli to develop psychological disorders, and we observed decreased FoxP3 and GATA3 mRNA in CRS. Nude mice, which are T cell-deficient mice, and their wild-type counterpart Balb/c were investigated for their vulnerability to stress and exhibition of depression-like behaviors. Both Balb/c and Balb/c Nude mice were randomly divided into two groups and one group was exposed to electric foot shock stress while the other remained as controls. Andehonic behavior of the mice was measured by sucrose preference test (SP) (a). Anxiety-like behavior was assessed using light dark test (LD) (b), and depressive-like behavior was assessed using tail suspension test (TST) (c). * p<0.05, ** p<0.01, n = 5-7 per group and the data shown are mean ± standard mean error (SEM).


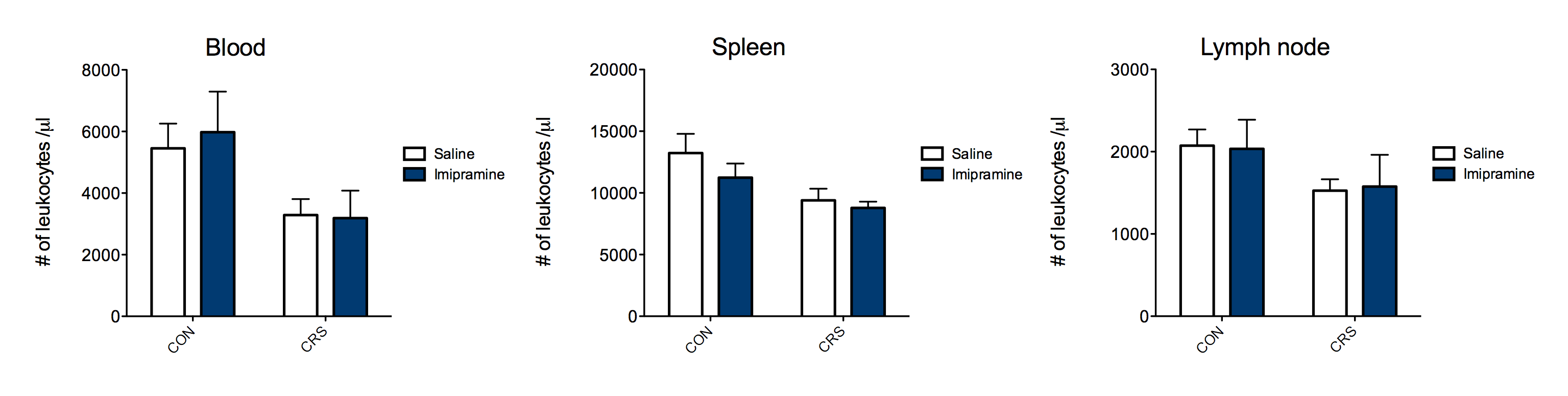


**S3. Effect of chronic restraint stress and imipramine treatment on leukocyte counts in the blood, spleen and lymph node.** Spleens and lymph nodes were removed and dissociated into single cells in RPMI with MACS dissociator (Miltenyi Biotec, Bergisch-Gladbach, Germany). Blood was collected from cardiac puncture using heparinized syringe and serum was collected. Then, red blood cells (RBCs) were removed with Pharm Lyse (BD Biosciences, San Jose, CA). Cells were further fixed with Cytofix (BD Biosciences) and resuspended in staining buffer (BD Biosciences) at 5 x10^6^/ml. Analysis was using a Becton-Dickinson FACS. CRS exposure decreased the leukocyte counts in the blood, spleen, and lymph node. However, imipramine co-treatment did not restore the leukocyte counts in all three sources. n = 3-5 per group and the data shown are mean ± standard mean error (SEM).
